# A Novel Reconstruction Technique During Pancreaticoduodenectomy After Roux-En-Y Gastric Bypass: How I do It

**DOI:** 10.1007/s11605-017-3405-2

**Published:** 2017-04-26

**Authors:** George Younan, Susan Tsai, Douglas B. Evans, Kathleen K. Christians

**Affiliations:** Pancreatic Cancer Program, Department of Surgery, Division of Surgical Oncology, Medical College of Wisconsin, 9200 W Wisconsin Ave., Milwaukee, WI 53226 USA

**Keywords:** Pancreaticoduodenectomy, Gastric bypass, Gastric remnant, Pancreaticogastrostomy

## Abstract

The altered anatomy in patients after bariatric surgery who have undergone a Roux-en-Y gastric bypass may pose a technical challenge for surgical removal of the pancreatic head. We treat patients with pancreas cancer with multimodality therapy in a neoadjuvant fashion followed by pancreaticoduodenectomy (PD). In patients with Roux-en-Y gastric bypass anatomy, the gastric remnant is preserved and used for pancreaticogastrostomy reconstruction and subsequently drained by the same jejunal limb used for the hepaticojejunostomy. This method of reconstruction takes advantage of the previous surgically altered anatomy and avoids the morbidity of a gastric remnant resection at the time of PD.

## Introduction

Bariatric procedures including Roux-en-Y gastric bypass (RYGB) are currently the most effective means for durable weight loss in the severely obese 1. Gastric bypass surgery has been proven to provide major health benefits and to decrease the overall risk of cancer and cancer-related death in surgically treated patients compared to obese non-operated patients 2, 3. Although the risk of developing pancreas cancer after a gastric bypass is low, the increased number of gastric bypass procedures being performed and the rising incidence of pancreas cancer make possible this unlikely combination of events 4.We present a novel approach to pancreaticoduodenectomy (PD) reconstruction in pancreatic adenocarcinoma patients who have undergone a previous Roux-en-Y gastric bypass.

### Resection Technique in PD in the Setting of RYGB

**Fig. 1:**
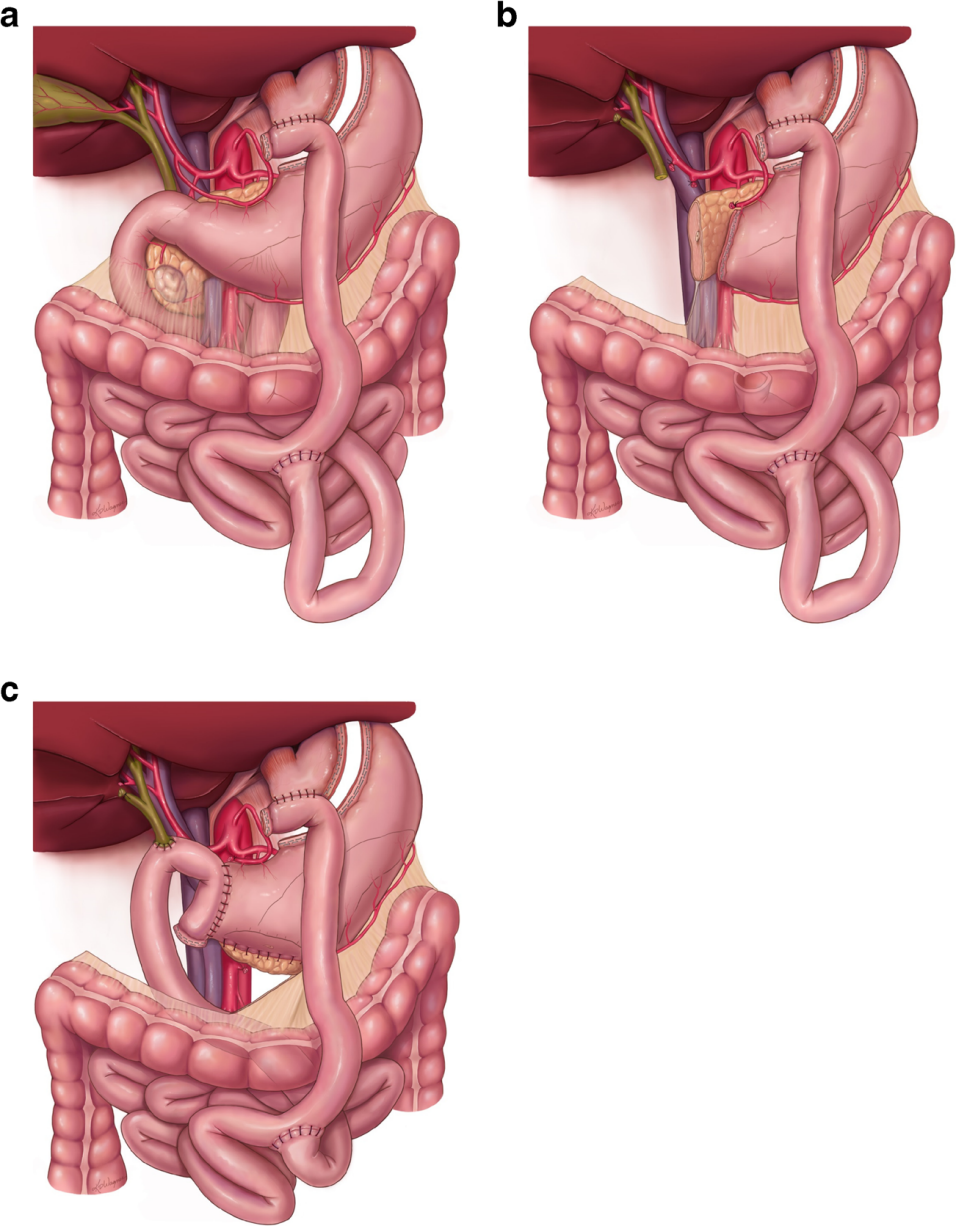
**a** RYGB anatomy prior to PD; a tumor is seen in the head of the pancreas. **b** The resulting anatomy post PD specimen removal; the jejunal limb is proximal to the previous jejunojejunostomy and is used for the reconstruction. **c** the PD anatomy after completion of the reconstruction with a PG; the jejunal limb is brought in a retrocolic fashion and used for the biliary and gastric remnant drainage

Laparoscopy is performed routinely prior to laparotomy to confirm the absence of metastatic disease. The operation is then completed as follows:

Step 1. The greater omentum is mobilized off of the transverse colon and the posterior gastric wall adhesions to the anterior pancreas are incised, widely opening the lesser sac. The hepatic flexure is mobilized from its retroperitoneal attachments. In the setting of prior RYGB, dense post-surgical adhesions are often encountered involving the gastric remnant. Any previous dissection in this region makes it technically more challenging to expose the infrapancreatic superior mesenteric vein (SMV). If possible, the visceral peritoneum along the inferior pancreatic border is incised beginning on the patient’s left of the middle colic vessels and carried out medial to lateral (right) and inferiorly, exposing the junction of the middle colic vein and SMV. The middle colic vein may enter directly into the anterior SMV or arise as a common trunk with the gastroepiploic vein (gastrocolic trunk). If they share a common trunk, the entire trunk may often need to be divided, or the common trunk preserved (as well as the middle colic vein) and the gastroepiploic vein divided; otherwise, the gastroepiploic vein is not divided until later in the operation when the pancreas is transected. In patients of very large BMI who have deep abdomens, the middle colic vein is often divided proximal to its junction with the SMV to avoid traction injury. It is also important to divide the middle colic vein when dealing with larger tumors or tumors with inferior extension from the uncinate process. In such cases, the base of the transverse colon mesentery and anterior leaf of small bowel mesentery is left attached to the tumor and resected en bloc with the pancreatic head. With extensive inflammation or scarring at the mesenteric root, the SMV may be exposed during step 6 when the pancreas is divided in a caudal direction beginning at the level of the portal vein (PV), which, by necessity, occurs without proximal control. This should only be done by very experienced pancreatic surgeons used to dealing with complex resections, especially in reoperative settings.
Step 2. The inferior vena cava (IVC) is identified and a Kocher maneuver is performed elevating all fatty and lymphatic tissue medial to the right gonadal vein and anterior to the vena cava along with the pancreatic head and duodenum. The Kocher is continued to the left side of the aorta, exposing the anterior surface of the left renal vein. The leaf of visceral peritoneum that is posterior to the pancreatic head is divided as it extends from the retroperitoneum to the right lateral aspect of the root of the small bowel mesentery.
Step 3. The common hepatic artery (CHA) is exposed proximal and distal to the right gastric and gastroduodenal arteries (GDA) by resecting the large lymph node that lies directly anterior to the proximal CHA. The right gastric artery and subsequently the GDA, are then ligated and divided. Following division of the GDA, the hepatic (common-proper) artery can be mobilized off of the underlying PV. Cholecystectomy is then performed and the PV exposed prior to transection of the common hepatic duct (CHD) at its junction with the cystic duct. Following biliary transection, a gentle bulldog is placed on the transected hepatic duct to prevent bile from gaining access to the rest of the abdomen during the remainder of the resection. The anterior wall of the PV is then exposed but the PV is not extensively mobilized until step 6, after the stomach and pancreas have been divided.
Step 4. The PD following RYGB is initiated as discussed above and every effort is made to preserve the previous Roux-en-Y gastrojejunostomy 5. Figure 1a delineates the initial RYGB anatomy. As the right gastric, gastroduodenal, and gastroepiploic arteries need to be divided for PD, extra care is taken to preserve the left gastric artery as it represents the sole arterial blood supply to the defunctionalized gastric remnant. The stomach remnant is then divided so as to perform a modest antrectomy (at the level of the third or fourth transverse vein on the lesser curvature and at the confluence of the gastroepiploic veins on the greater curvature) using a linear gastrointestinal stapler (GIA, Ethicon Inc.). The omentum is then divided at the level of the greater curvature transection with the harmonic scalpel (Ethicon Inc., Somerville, NJ) or LigaSure device (Valleylab, Boulder, CO).
Step 5. The attachments of the ligament of Treitz are taken down carefully to avoid the inferior mesenteric vein (IMV). The pancreatobiliary limb from the previous RYGB is divided distal to the ligament of Treitz and if of adequate length (as is the case in a modern RYGB) it will be used later in the operation for biliary reconstruction. The mesentery of the proximal jejunum and distal duodenum is sequentially ligated with the LigaSure device (Valleylab, Boulder, CO). The duodenal mesentery is divided to the level of the aorta. This devascularized segment of duodenum and jejunum is then reflected beneath the mesenteric vessels into the right upper quadrant.
Step 6. Traction sutures are placed on the superior and inferior borders of the pancreas and the pancreas is transected with electrocautery at the pancreatic neck (directly overlying the PV-SMV). If there is tumor involvement of the PV or SMV, the pancreas is divided further distally (leftward) to prepare for segmental venous resection. The specimen is then separated from the SMV by ligation and division of the small venous tributaries to the uncinate process and pancreatic head. The uncinate process must be removed from the SMV to fully mobilize the SMV-PV confluence as well as to identify the SMA. If the SMV is not completely mobilized, it is difficult to expose the SMA and therefore the inferior pancreaticoduodenal vessels (IPDAs). Complete mobilization of the SMV involves identification of the jejunal branch of the SMV originating from the right posterolateral aspect of the SMV at the uncinate process, or less commonly, coursing anterior to the SMA. The jejunal branch normally gives off branches to the uncinate process which need to be divided. If tumor involvement of the SMV, at the level of the jejunal branch, prevents dissection of the uncinate process from the SMV, the jejunal branch is divided (made possible by a robust ileal branch). Once the uncinate process is separated from the distal SMV, medial retraction of the SMV-PV confluence allows exposure of the SMA. The specimen is then separated from the right lateral wall of the SMA which is then dissected to its origin at the aorta. Direct SMA exposure allows individual direct ligation of the IPDAs (usually two). Frozen sections are performed of the pancreatic and bile duct margins. Positive margins (malignancy, not dysplasia) mandate further resection until clear. The specimen must be oriented for the pathologist and the SMA margin identified and inked according to AJCC standards. Figure 1b delineates the resulting anatomy after PD in RYGB patients.

### Reconstruction Following PD in the Setting of RYGB

The reconstruction is different from our standard approach in the following ways:

- The pancreatic remnant is mobilized from the underlying splenic vein/artery and retroperitoneum for an appropriate distance to allow for a tension-free anastomosis. A two layer duct-to-mucosa pancreaticogastrostomy (PG) is performed to the posterior wall of the gastric remnant with monofilament absorbable sutures.
- The proximal jejunum just distal to the ligament of Treitz and proximal to the jejunojejunostomy is brought retrocolic (not retroperitoneal) into the right upper quadrant through a large incision made in the transverse colon mesentery to the left of the middle colic artery. Approximately 8–10 cm from the end of the jejunal limb, an end-to-side, single layer hepaticojejunostomy is constructed using interrupted monofilament absorbable suture. The end of the jejunal limb is then sewn to the anterior surface (or staple line) of the gastric remnant. This is done in an end-to-side or side-to-side fashion, thereby decompressing the gastric remnant and therefore, the pancreaticogastrostomy (Fig. 1c). Modern RYGB procedures are most commonly done in a fashion where there is enough length of small bowel proximal to the jejunojejunostomy to be used for our novel method of post-PD reconstruction. In the circumstance of a shorter small bowel segment proximal to the jejunojejunostomy, a new roux limb can be created distal to the jejunojejunostomy.
- In cases where the inferior mesenteric vein (IMV) drains directly into the superior mesenteric vein or superior mesenteric vein/portal vein (SMV-PV) confluence and the splenic vein requires ligation due to tumor encasement of the SMV-PV confluence, the IMV is not present to decompress the now ligated splenic vein. In the absence of a mechanism for splenic vein decompression, the patient is at risk for sinistral portal hypertension and gastrointestinal varices. In these circumstances, we create a splenorenal shunt with an end-to-side anastomosis between the splenic vein and the left renal vein 6, 7. This shunt is of particular importance in RYGB patients where the short gastric veins have been previously divided. Splenic vein ligation is also used when additional exposure to the proximal celiac artery or superior mesenteric artery may be necessary and the vein is tethered to the tumor or more significantly encased.

## Discussion

Weight loss surgery has gained significant popularity over the past few decades and has been proven to be the most durable medical approach to weight loss and the associated morbidities of obesity 8, 9. Prior to the current popularity of laparoscopic sleeve gastrectomy, laparoscopic gastric bypass was the most commonly used procedure 10. With the increased number of patients who have a history of RYGB and the increased detection of pancreatic lesions on cross sectional imaging, surgeons may be confronted with this unusual combination of pathology. This represents a complicated anatomic situation due to altered intestinal anatomy and the potential for compromised nutritional status if a PD is performed 11, 12.

A RYGB procedure is most commonly done laparoscopically and involves creating a small gastric pouch by dividing the upper part of the fundus of the stomach thereby leaving a large gastric remnant in situ. A Roux limb of jejunum is then brought in an ante- or retro-colic fashion and anastomosed to the small gastric pouch. A jejunojejunostomy is constructed by anastomosis of the pancreatobiliary limb to the distal jejunum 13. Figure 1a illustrates the most common anatomy following RYGB.

There is little published literature discussing PD for pancreas cancer following RYGB. Most published articles involve small series of patients or case reports (Table 1). Swain et al. reported a series of five patients who underwent PD for pancreatic or ampullary tumors and suggested a method of reconstruction which included resection of the gastric remnant. The gastric remnant in one case could not be technically resected due to dense adhesions 17. This was also the case in other reports where left upper quadrant adhesions precluded a safe resection of the gastric remnant due to the risk of injury to the functional gastric pouch and the previously created gastrojejunostomy 18, 19. A separate Roux limb has been proposed to drain the gastric remnant when it could not be resected. All other case reports describe a technique of reconstruction involving resection of the gastric remnant 20–22. Pancreaticojejunostomy (PJ) was the only technique used for pancreatic reconstruction in all reported series and if the gastric remnant could not be resected, an additional Roux limb configuration was preferred.

**Table 1:** Summary of published series to date on PD after gastric bypass

Study	#Patients	Gastric Remnant	Pathology	Reconstruction
Khithani et al., 14	2	0	PDAC	PJ
Rutkoski et al., 19	1	1	PDAC	PJ
Swain et al., 15	5	1	PDAC, AC, PNETx2	PJ
Nikfarjam et al., 21	2	0	Benign CBD stricture	PJ
de la Cruz-Muñoz et al., 22	1	0	PNET	PJ
Theodoropoulos et al., 18	1	1	PDAC	PJ
Helmick et al., 16	2	2	IPMN, Benign/fibrosis	PJ

*PDAC* pancreatic ductal adenocarcinoma, *AC* ampullary carcinoma, *PNET* pancreatic neuroendocrine tumor, *IPMN* intraductal papillary mucinous neoplasm, *PJ* pancreaticojejunostomy

PD is one of the most complex abdominal operations; mastery of surgical technique and significant technical experience in pancreas surgery are required for optimal outcomes 5, 23. Performing a remnant gastrectomy during PD adds significant complexity and time to an already long procedure, especially in a reoperated surgical field where left upper quadrant adhesiolysis might result in injury to the gastric pouch and/or the gastrojejunostomy. Our technique is therefore based on keeping the gastric remnant in situ. Providing adequate drainage of the gastric remnant requires some form of gastrojejunostomy involving the gastric remnant. The jejunal limb proximal to the jejunojejunostomy is used for the biliary reconstruction and for drainage of the gastric remnant; a duct-to-mucosa PG is created to the posterior wall of the gastric remnant. We believe that this method of reconstruction provides an efficient way to decompress the gastric remnant which does not need to be resected during reconstruction.

We used a PG rather than a PJ for multiple reasons. Because we preserved the remnant stomach and drained it through the same jejunal limb used for biliary reconstruction, it was technically easier to create an anastomosis between the pancreas remnant and the posterior wall of the stomach than to add a third anastomosis to the jejunal limb. This also avoids unnecessary tension that could result from anastomosing both the gastric and the pancreas remnants, through separate anastomoses, to the jejunal limb. PG has theoretical benefits compared to PJ as a reconstruction method following PD 24. A tension-free anastomosis is one potential benefit as the pancreas remnant is in close proximity to the posterior gastric wall. In addition, with a PG, the pancreas is anastomosed to a thick, richly vascularized gastric wall, theoretically decreasing the chance of anastomotic leak or fistula. Other theoretical benefits include the failure of activation of pancreatic enzymes in the absence of enterokinase and when in direct contact with the acidic gastric environment; another mechanism to potentially decrease the risk of pancreatic fistula 25. Finally, an additional benefit to this form of reconstruction is that the gastric remnant is defunctionalized and not in continuity with the gastrointestinal tract. The alternative methods of drainage of the gastric remnant suggested in the literature used an additional jejunal limb (to the retained stomach remnant) for this purpose, resulting in a potentially ulcerogenic anatomy, especially when a retained antrum is a part of the gastric remnant 19. In contrast, drainage of the gastric remnant through the same jejunal limb used for biliary drainage, combined with a PG, will have a protective effect against the formation of a marginal ulcer. In summary, we have demonstrated that the use of PG is safe and effective for post-PD reconstruction in patients who have a remote history of a RYGB 24, 26, 27.

## Conclusion

PG in the setting of pre-existing RYBG anatomy is an attractive method to efficiently take advantage of the previous surgical procedure. The morbidity of and additional procedure time associated with resection of the gastric remnant are thereby avoided.
